# CTX-M-27–Producing *Escherichia coli* of Sequence Type 131 and Clade C1-M27, France

**DOI:** 10.3201/eid2305.161865

**Published:** 2017-05

**Authors:** André Birgy, Philippe Bidet, Corinne Levy, Elsa Sobral, Robert Cohen, Stéphane Bonacorsi

**Affiliations:** Institut National de la Santé et de la Recherche Médicale, Paris, France (A. Birgy, P. Bidet, S. Bonacorsi);; Université Paris Diderot, Paris (A. Birgy, P. Bidet, S. Bonacorsi);; Hôpital Universitaire Robert-Debré, Paris (A. Birgy, P. Bidet, S. Bonacorsi);; Association Clinique Thérapeutique Infantile du Val de Marne, Saint Maur des Fossés, France (C. Levy, E. Sobral, R. Cohen);; Groupe de Pathologie Infectieuse Pédiatrique, Paris (C. Levy, R. Cohen);; Centre Hospitalier Intercommunal de Créteil, Créteil, France (C. Levy, R. Cohen);; Université Paris-Est Créteil, Créteil (C. Levy, R. Cohen)

**Keywords:** *Escherichia coli*, *E. coli*, ST131, C1-M27 clade, CTX-M-27, ESBL, *fimH*30, *fimH*41, children, fecal carriage, France, bacteria

**To the Editor:** We read with great interest the Matsumura et al. paper describing extended-spectrum β-lactamase (ESBL) CTX-M-27–producing *Escherichia coli* of sequence type (ST) 131 clonal group, an emerging clade called C1-M27 (*1*). ST131 *E. coli* having *bla*_CTX-M-27_ has been described in several countries. We observed an alarming increase of this clonal group in the fecal carriage of children in France (0% in 2010 to 65% in 2015 among ESBL-producing ST131 *E. coli*) (*2*). 

We wondered whether this clonal group’s expansion in France was attributable to the same clade (C1-M27). For that, we designed primers (M27PP1-B-F, 5′-TTACTCCGACTATGCGTTCAC-3′; M27PP1-B-R, 5′-CAAACTTGCCCCTGATAGCG-3′; amplicon length, 1.5 kb) to amplify the insertion site of the structure comprising the direct repeat and prophage-like genomic island of *E. coli* PCN033, as previously described (*1*). PCR was performed on our recently described collection of 39 ESBL-producing ST131 *E. coli*, including 16 CTX-M-27–producing *E. coli*: 13 of subgroup O25b with *fimH*30 allele and 3 of O16 subgroup with *fimH*41 allele (*2*). Results showed that 81% (13/16) of the CTX-M-27–producing *E. coli* ST131 had the M27PP1 structure, similar to strain PCN033, and thus belong to the C1-M27 clade. Therefore, the C1-M27 clade found in Asia and America is also present in Europe in the fecal flora of young children. The 3 isolates belonging to the O16 subgroup with *fimH*41 lacked M27PP1, suggesting that *bla*_CTX-M-27_ might diffuse among non-H30 ST131 *E. coli* without this prophage-like genomic island. Finally, the non–CTX-M-27–producing ST131 *E. coli* of our collection were negative for M27PP1 elements.

Our results show that CTX-M-27–producing *E. coli* ST131 subgroup O25b with *fimH*30 allele (one third of the ESBL-producing ST131 carriage isolates) isolated from children in France belong to C1-M27 and that CTX-M-27–producing O16 strains display distinct genetic characteristics. Altogether, our data confirm the worldwide distribution of C1-M27 and its high prevalence in children in France.
